# Plasma imatinib levels and *ABCB1* polymorphism influences early molecular response and failure-free survival in newly diagnosed chronic phase CML patients

**DOI:** 10.1038/s41598-020-77140-9

**Published:** 2020-11-26

**Authors:** Bharathi M. Rajamani, Esther Sathya Bama Benjamin, Aby Abraham, Sukanya Ganesan, Kavitha M. Lakshmi, Senthamizhselvi Anandan, Sreeja Karathedath, Savitha Varatharajan, Ezhilpavai Mohanan, Nancy Beryl Janet, Vivi M. Srivastava, Shaji Ramachandran Velayudhan, Uday P. Kulkarni, Anup J. Devasia, N. A. Fouzia, Anu Korula, Biju George, Alok Srivastava, Vikram Mathews, Poonkuzhali Balasubramanian

**Affiliations:** Department of Haematology, Christian Medical College, Vellore, 632004 India

**Keywords:** Cancer, Medical research

## Abstract

Achieving early molecular response (EMR) has been shown to be associated with better event free survival in patients with chronic phase chronic myeloid leukemia (CP-CML) on Imatinib therapy. We prospectively evaluated the factors influencing the 2-year failure free survival (FFS) and EMR to imatinib therapy in these patients including day29 plasma Imatinib levels, genetic variants and the gene expression of target genes in imatinib transport and biotransformation. Patients with low and intermediate Sokal score had better 2-year FFS compared to those with high Sokal Score (p = 0.02). Patients carrying ABCB1-C1236T variants had high day29 plasma imatinib levels (P = 0.005), increased EMR at 3 months (P = 0.044) and a better 2 year FFS (P = 0.003) when compared to those with wild type genotype. This translates to patients with lower ABCB1 mRNA expression having a significantly higher intracellular imatinib levels (P = 0.029). Higher day29 plasma imatinib levels was found to be strongly associated with patients achieving EMR at 3 months (P = 0.022), MMR at 12 months (P = 0.041) which essentially resulted in better 2-year FFS (p = 0.05). Also, patients who achieved EMR at 3 months, 6 months and MMR at 12 months had better FFS when compared to those who did not. This study suggests the incorporation of these variables in to the imatinib dosing algorithm as predictive biomarkers of response to Imatinib therapy.

## Introduction

Imatinib mesylate, a tyrosine kinase inhibitor (TKI), has revolutionized the treatment of chronic myeloid leukemia (CML) changing it from a life-threatening disease to a condition that can be controlled in the vast majority of cases by oral medication. For those patients who develop resistance or intolerance to imatinib, choices of second or third generation TKIs are available. However, responses are the best when such changes in therapy are done prior to disease progression to an accelerated phase or blast crisis. This decision process on continuing therapy or changing TKI is compounded by the high cost of these second generation drugs in a predominantly self-paying system as exists in our country. Identifying the sub-optimal responders and early switch of therapy is essential since the achievement of major molecular response (MMR) or deep molecular response (DMR) within 3–6 months post TKI therapy has been shown to significantly improve progression free survival^1–4^.

Several factors have been reported to contribute to sub-optimal response or resistance to imatinib^5,6^. Among the *BCR-ABL1* dependent mechanisms, development of mutations in the kinase domain of *BCR-ABL1*^7–9^ plays a major role. Several imatinib independent mechanisms of resistance have also been reported including overexpression of efflux transporters^10–12^, decreased expression of influx transporters^13–15^, decreased plasma levels^16,17^, binding to plasma proteins^18^, and genetic polymorphisms in the enzymes and transporters involved in imatinib transport or biotransformation^19,20^. With the exception of one study reporting an intronic deletion polymorphism in the *BIM* (*BCL2Like11*) gene to be associated with imatinib resistance^21–23^, there are no other prospective studies in patients with CML of Asian origin where all the parameters have been systematically evaluated. This is important due to the ethnic differences in genetic polymorphisms, which in turn could influence the differences in systemic exposure to anticancer drugs^24,25^.

Although there are several studies^15,17,21,22,26^ exploring the factors influencing MMR and DMR in the international scale, there is no comprehensive analysis of the factors influencing EMR and MMR which in turn influence failure free survival (FFS). Our aim was to prospectively document response to imatinib as first line therapy using serial molecular monitoring to document EMR and MMR to evaluate the factors influencing response to imatinib therapy in newly diagnosed patients with CML-CP.

## Patients and methods

### Patients

All newly diagnosed, imatinib naïve adult CP- CML patients visiting Department of Haematology, Christian Medical College, Vellore were enrolled in the study after obtaining written informed consent. Ethical approval for this study has been approved by Institutional review board (IRB Min no: IRB (EC)-4–11-06–2008 dated June 19, 2008) at Christian medical college, Vellore -632,004, Tamilnadu, India. All methods in the study were performed in accordance with relevant guidelines and regulations.

### Morphology/RT-PCR/Cytogenetics/FISH analysis

All patients at diagnosis were analyzed for the presence of *BCR-ABL1* fusion gene by Fluorescence in-situ hybridization (FISH). Interphase FISH analysis was performed using fixed cell suspensions obtained by direct or unstimulated overnight cultures of peripheral blood or bone marrow as reported previously^27^. Although karyotyping is not routinely performed for CML patients in our centre, it was done in those patients who gave consent for bone marrow aspiration. Peripheral blood was collected at diagnosis and RNA was extracted using Trizol method. cDNA was synthesized from 2 μg RNA using random hexamers and reverse transcriptase enzyme (High capacity c-DNA synthesis kit, Thermo Scientific) followed by RT-PCR to identify the *BCR-ABL1* fusion transcript [e13a2/e14a2 or e1a2).

### Plasma and intracellular levels of Imatinib

Blood samples were collected on day29 post imatinib therapy in EDTA anticoagulated tubes and plasma was separated and stored immediately. Trough plasma imatinib and desmethyl imatinib (imatinib metabolite) concentration was assessed using HPLC (High Performance Liquid Chromatography) Ultra-violet detection method as reported previously^28^ with minor modifications. Dasatinib was used as an internal standard.

Peripheral blood mononuclear cells (PBMNCs) (15*10^6^/mL) from CML patients at diagnosis was incubated at 37 °C for 24 h and treated with 5 μM imatinib for 5 h. The cells were washed with phosphate buffered saline (PBS) and the cell pellets were stored at − 80 °C until analysis. Just before processing, the samples were thawed, the cells were lysed by sonication for 10 min on ice. Intracellular levels of Imatinib were analyzed by the same method as that of plasma imatinib incorporating minor changes from a previously published protocol^28^.

### BCR-ABL1 molecular monitoring

Peripheral blood was collected at diagnosis, 3, 6 9, 12 and 18 months’ post imatinib therapy to check the *BCR-ABL1* transcript level. Additional samples were analyzed depending on clinical needs. The *BCR-ABL1* transcript levels were quantified using real time quantitative reverse transcriptase polymerase chain reaction (qRT-PCR) method as reported previously^29^.

### BCR-ABL1 kinase domain mutation detection

Mutations in the *BCR-ABL1* kinase domain were evaluated in patients with suboptimal response as reported previously^30^. The sequences were aligned using SeqScape (Applied Biosystems) and the type of the mutation was predicted using Mutation Taster (https://www.mutationtaster.org/).

### RNA expression of influx and efflux transporters

Total RNA was extracted using Trizol method and 1–2 μg RNA was used for c-DNA synthesis (High capacity cDNA Reverse Transcription Kit, Applied Biosystems). RNA expression of various Imatinib efflux and influx transporters were analyzed using TaqMan based assays [assay IDs: *ABCB1* (HS01067802-m1), *ABCG2* (HS01053787-m1), *SLC22A1* (HS00427554-m1), *ABCA3* (HS00184543-m1), *ABCA5* (HS00363322-m1), *ABCA6* (HS00365329-m1), *ABCB5* (HS02889060-m1), *ABCB6* (HS00180568_m1), *ABCB7* (HS00188776-m1), *ABCB8* (HS00894817-m1), *ABCB10* (HS00429240-m1), *ABCB11* (HS00184824-m1), *ABCC1* (HS00219905-m1), *ABCC3* (HS009784173-m1), *ABCC4* (HS00988717-m1), *ABCC11* (HS01090768-m1)] and the expression was normalized to the housekeeping gene *GAPDH* (4352934E).

### RNA expression of influx and efflux transporters in CD34^+^ fraction

CD34^+^ cells were enriched from the CML patient sample and healthy donors using Easysep magnetic enrichment kit (https://www.stemcell.com/easysep-human-cd34-positive-selection-kit-ii.html). RNA extraction, c-DNA synthesis and expression of influx (*hOCT1*) and efflux (*ABCB1* & *ABCG2*) transporter analysis was also done as explained previously.

### Polymorphisms in imatinib transporters and drug metabolizing enzymes

Genomic DNA was extracted from peripheral blood using standard phenol–chloroform method and 50-100 ng DNA was used for each PCR. All coding exons with flanking introns were screened for *SLC22A1* and known single nucleotide polymorphisms (SNP) in Imatinib efflux transporters *ABCB1* (exon 26), *ABCG2* (promoter, exon 2 and exon 5), *OCTN1*/*SLC22A4* (exon 9) were screened by PCR followed by sequencing and variants were analyzed by SeqScape software. Three SNPs in Cyp3A4/3A5 were analyzed by restriction fragment length polymorphism (RFLP). The primer sequences and the source of the methods are listed in Supplementary Table 1.

### *BIM* deletion polymorphism

Genomic DNA was used to assess the *BIM* deletion polymorphism by PCR as previously reported^21^. The PCR conditions used were: 95 °C for 10 min and 40 cycles of 94 °C for 30 s, 60 °C for 30 s, 72 °C for 10 min. Emerald Master mix (TaKaRa BIO Inc., Shiga, Japan) was used for this PCR. The amplified products when subjected to 3% agarose gel electrophoresis showed the band size of 216 bp for wild-type and 173 bp if there was deletion.

### *GSTT1*, *GSTM1* deletions, GSTA1*B and GSTP1*B polymorphisms

*GSTT1* and *GSTM1* deletion, GSTA1*B and GSTP1*B ([Ile105val] rs1965) polymorphisms were screened by methods as reported previously^31^. Primers used for PCR reaction and the conditions are listed in supplementary Table 1.

### Assessment of response and definition of outcome

Response criteria including complete hematological response (CHR) and molecular response were defined according to the European Leukaemia Net (ELN) guidelines^32^. Hematologic response was defined as normalized peripheral blood cell counts (WBC < 10 X10^9^/L and platelet count < 450 X10^9^/L) without evidence of peripheral blasts, promyelocytes, or myelocytes, and without evidence of extramedullary disease including disappearance of palpable splenomegaly lasting for at least 4 weeks. Molecular response was classified based on *BCR-ABL1* to control gene transcript ratios, expressed on the International Scale (IS) as reported previously from our lab^29^. EMR was defined as *BCR-ABL1* to control gene ratio of ≤ 10% in the IS at 3 and ≤ 1% at 6 months; MMR was defined as *BCR-ABL1* to control gene transcript ratio of ≤ 0.1% in the IS, 12 months after imatinib therapy. In patients without an MMR at 12 months, mutation analysis of the *BCR–ABL1* fusion transcript was performed by direct sequencing.

Patients were classified as intolerant to imatinib therapy if the patient meets one or more of the criteria as reported previously^33^. Patients who progressed to accelerated phase or blast crisis were classified as non-responders at all-time points. FFS was assessed by defining an event as: loss of hematological or cytogenetic response, progression to advanced phase or blast crisis, stem-cell transplant, death from any cause, change in imatinib dose or switch to a second generation TKI due to suboptimal response, as reported previously^34^. Overall survival (OS) was calculated from the initiation of imatinib therapy until the date of death from any cause or the date of last follow-up.

### Statistical analysis

All statistical analyses were performed using SPSS (IBM SPSS statistics version 21.0, Armonk, NY) and GraphPad Prism 5 (GraphPad Software, La Jolla, CA; www.graphpad.com) software; *p* value < 0.05 was used for significance testing. Genotype distribution was tested for Hardy–Weinberg equilibrium (HWE). Comparison of RNA expression levels of candidate genes was done using one-way ANOVA and student’s t-tests. For analyzing PFS, patients who stopped imatinib or switched treatment during follow-up were censored at the time of stopping or switching.

## Results

### Patient demographics

The demographics of the CP-CML patients enrolled in this study are listed in Table 1. There were 108 males and 52 females with a median age of 36 (range 18–65) years. One hundred and ten patients had low or intermediate Sokal score while 50 patients had high Sokal Score. Each patient started with an oral dose of 400 mg of imatinib daily. The median follow-up of the patients post therapy was 83 months (range: 12 to 120 months). Of the 146 patients in whom karyotyping was done, 128 had only t(9;22) and 18 had the t(9;22) with additional chromosomal abnormalities.

**Table 1:** Patient Demographics (n = 160 CP-CML).

Age	Median (Range) : 36 years (18–65)
Sex	Males: 108; Females: 52
Sokal Score	Low (< 0.8) N = 49
	Intermediate (0.8–1.2) N = 61
	High (> 1.2) N = 50
Median follow-up (range)	83 (12–120 months)
Karyotype at diagnosis	Only 9;22 n = 128
	t(9;22)with additional chromosome abnormalities n = 18
	Not available N = 14
**BCR-ABL1 Fusion transcript**	
e13a2 type	51
e14a2 type	109
**Drug**	
Glivec	140
Veenat	20

### Response to imatinib therapy

One hundred and fifty of the 160 patients (94%) achieved complete hematological response at 3 months. The proportion of patients who achieved EMR at 3, 6 months and MMR at 12 months’ post imatinib treatment is listed in Table 2. Neither the *BCR-ABL1* transcript type nor Sokal score showed significant association with achieving EMR/MMR. The rate of FFS at 24 months’ post imatinib therapy in these patients was 78 ± 3.3%. The events included switch of TKI (n = 18), escalation (n = 33) of imatinib dose due to in-ability to afford or access 2nd/3rd generation TKI, palliative therapy due to severe toxicity (n = 7), imatinib dose reduced due to intolerance (n = 5), stem cell transplantation (n = 1) and progressive disease (n = 3) requiring chemotherapy or death. The decision to switch TKI or change the dose of TKI was entirely upto the discretion of the treating doctor as per the department policy and ELN guidelines.

**Table 2:** Incidence of early molecular response (EMR) at 3 & 6 months and major molecular response (MMR) status at 12 months in CML-CP patients on imatinib therapy.

	3 months		6 months		12 months	
Molecular response*	< 10% (66/115; 57%)		< 1% (66/135; 49%)		< 0.1% (61/145; 42%)	
	e13a2	e14a2	e13a2	e14a2	e13a2	e14a2
	17	49	18	48	36	25
Transcript type#	> 10% (49/115; 43%)		> 1% (69/135; 51%)		> 0.1% (84/145; 58%)	
	e13a2	e14a2	e13a2	e14a2	e13a2	e14a2
	17	32	24	45	61	23

*Denominators represent the number of patients for whom basal data was available at scheduled time point & those who were still on imatinib at that time.

^#^Transcript type not associated with molecular response at 3, 6 and 12 months.

### Spectrum of *BCR-ABL1* kinase domain mutation in imatinib non-responders

In patients who failed to achieve milestone responses to imatinib therapy, presence of mutation in the *BCR-ABL1* kinase domain was tested. Of the 80 patients tested, 22 patients had mutations in the *BCR-ABL1* kinase domain. The spectrum of *BCR-ABL1* kinase domain mutation identified in these patients is listed in Supplementary Table 2. T315I and G250E were the common mutations identified.

### Genotype/Allele frequencies of genetic variants

The allele frequencies of the 29 genetic variants in 12 genes [drug transporter genes *SLC22A1*, *ABCB1* & *ABCG2* and drug metabolizing enzyme genes GST, *CYP3A4/A5*] screened in this patient cohort is listed (Supplementary Table 3). The genotypes of all the genetic variants were in Hardy–Weinberg equilibrium. The *SLC22A1* variants were also screened in normal healthy volunteers (n = 100), as there was no Indian data at the time we started this study (Supplementary Table 4). The allele frequencies of *SLC22A1* variants were comparable between patients and normal controls. The *SLC22A1* exon5 variant (Arg287Gly) was in complete linkage disequilibrium with an exon 6 variant (Thr340Met). An 8 bp ins polymorphism in intron7 was in complete linkage disequilibrium with an exon7 coding variant (Met408Val; rs628031) which results in a splice variant. When we tried to amplify the full length of *SLC22A1*cDNA in patients with the intron 7 ins polymorphism in the mutant state, there was a truncated transcript but no full length *SLC22A1* transcript while the heterozygotes showed both truncated and full length transcripts; the samples with wild type genotype showed only the full length transcript upon RT-PCR.

### RNA expression of imatinib influx transporter *hOCT1* and ABC transporters

Expression of Imatinib influx (*hOCT1*/*SLC22A1*) and efflux transporters (*ABCB1, ABCG2*, SLC22A1, *ABCA3*, *ABCA5*, *ABCA6*, *ABCB5*, *ABCB6*, *ABCB7*, *ABCB8*, *ABCB10*, *ABCB11*, *ABCC1*, *ABCC3*, *ABCC4* and *ABCC11*) showed wide interpatient variability (Supplementary Table 5).

### Plasma and intracellular imatinib and desmethyl imatinib levels

In this cohort, 87.5% of patients (n = 140) were on Glivec and 12.5% patients (n = 20) were on Veenat, a generic imatinib formulation from India. The trough plasma imatinib and desmethyl imatinib levels on day29 was available only in 67 patients. This is due to reasons such as patients not visiting the clinic by the end of one month, taking imatinib at night so trough level sampling was not possible or have withheld imatinib due to intolerance. There was no significant difference in trough plasma imatinib levels between patients receiving Glivec vs. generic imatinib. The median plasma imatinib and desmethyl imatinib concentrations on day29, were 1050 ng/mL (106–5035 ng/mL) and 191 ng/mL (31–2161 ng/mL) respectively. The median intracellular imatinib level after ex-vivo incubation of primary CML cells (n = 64) with imatinib was 1225 ng/mL (range 181-12848 ng/mL).

### Plasma imatinib levels influence EMR and MMR to imatinib therapy

**Figure 1:**
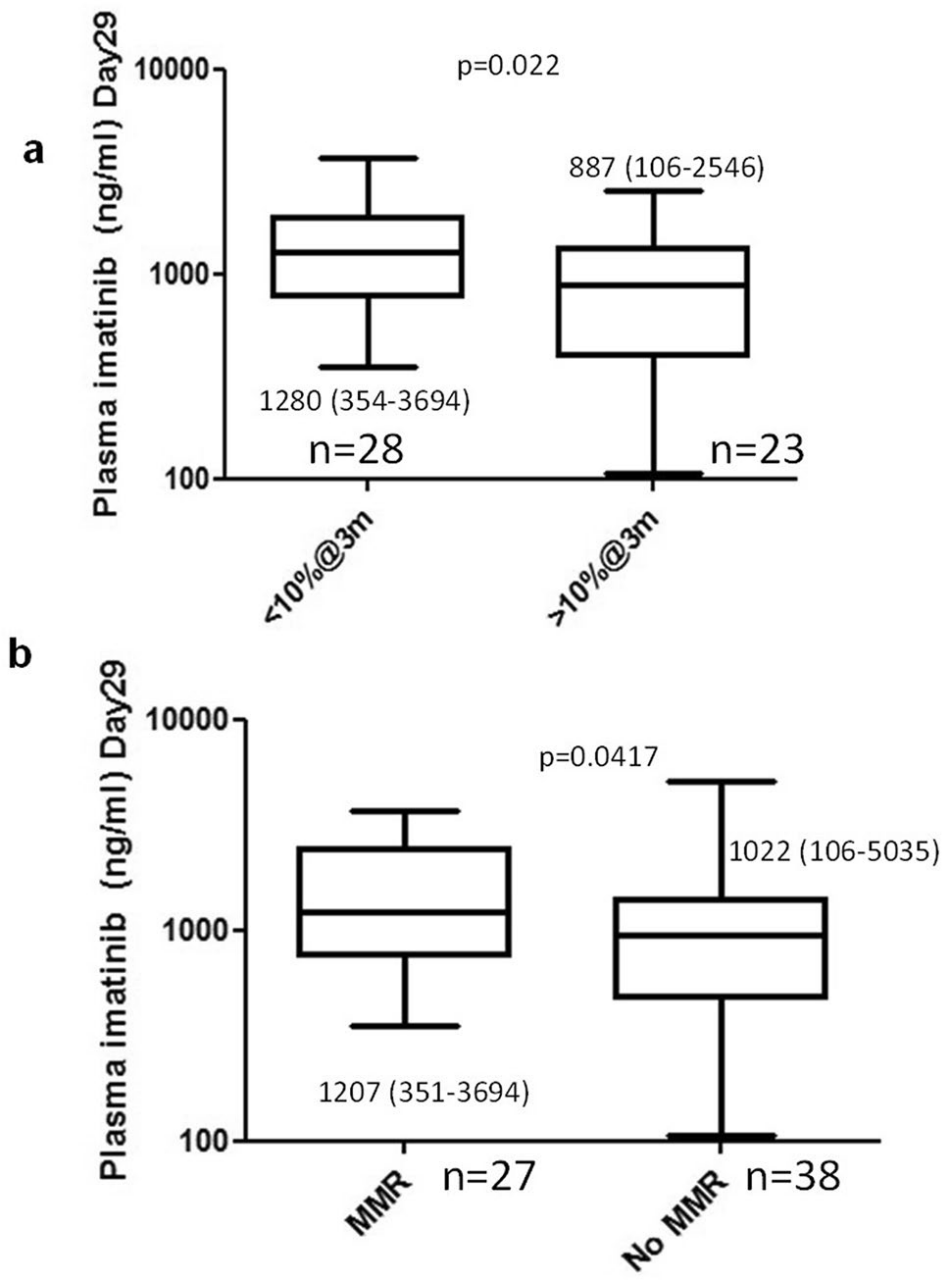
Plasma imatinib levels predict early molecular response at 3 & 6 months and MMR at 12 months post imatinib therapy. (**a**) Plasma imatinib levels (median, range) on day29 with early molecular response at 3 months and (**b**) in patients with and without MMR to imatinib at 12 months. Statistical significance was calculated using Mann–Whitney U test.

The median plasma imatinib level on day29 was significantly higher in those who achieved EMR at 3 months compared to those who did not (1280 ng/ml vs 887 ng/ml; p = 0.022 Fig. 1a). The median plasma imatinib levels on day29 was significantly higher in those who achieved MMR at 12 months compared to those who did not (1207 ng/ml vs 1022 ng/ml; p = 0.0417 respectively; Fig. 1b).

### Plasma Imatinib levels are influenced by genetic variants in *ABCB1*

**Figure 2:**
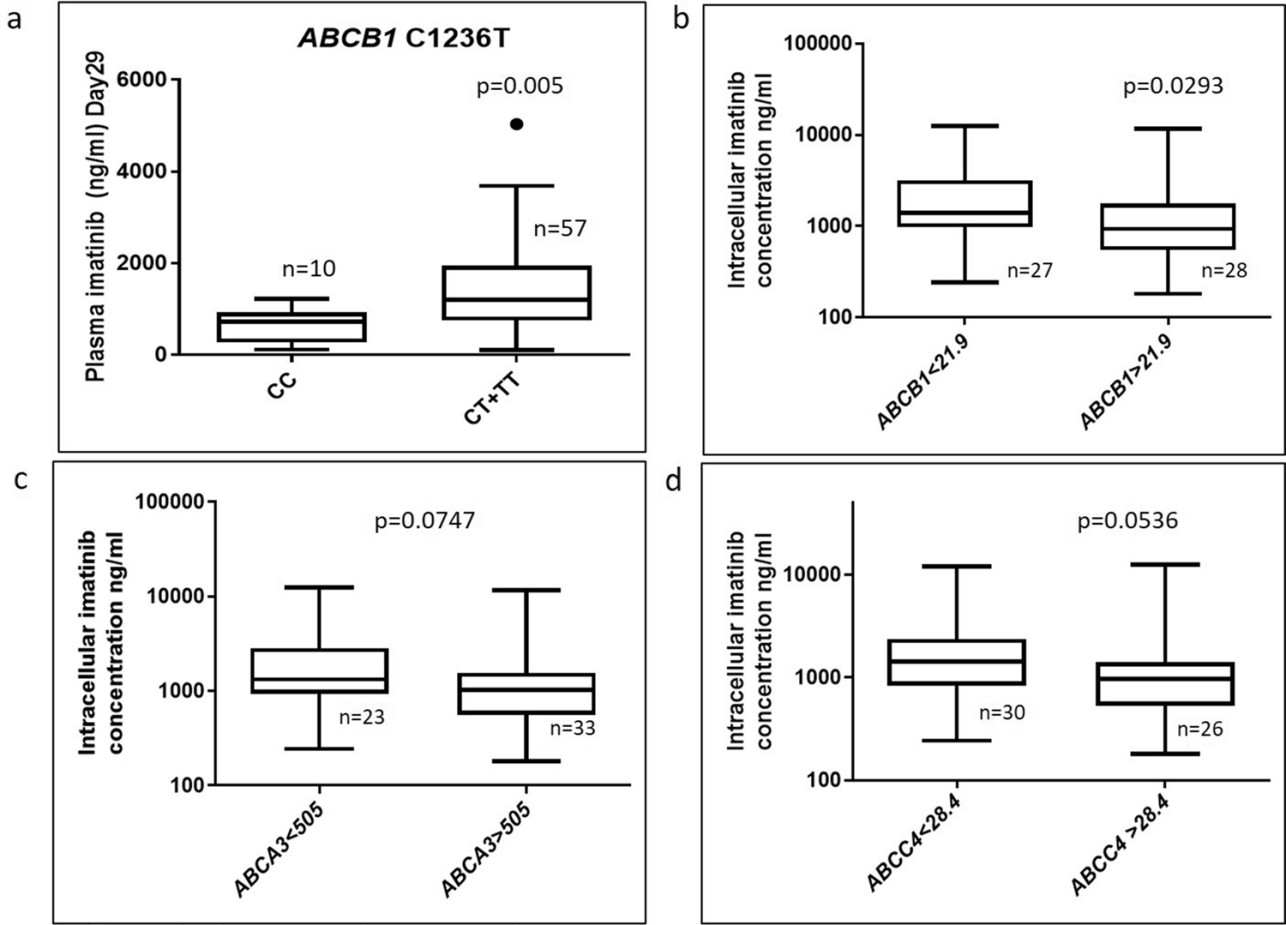
*ABCB1* polymorphism influences plasma imatinib levels and RNA Expression of ABC transporters influence intracellular Imatinib levels (b) Plasma imatinib levels on day29 post imatinib therapy in patients with *ABCB1*/*MDR1* C1236T genotypes (wt (CC) n = 10; Het + Mut (CT + TT) n = 57). (**b**) Intracellular imatinib levels in patients with above or below median expression of *ABCB1* (median expression-21.9), (**c**) *ABCA3* (median expression-505 below median n = 23 vs above median n = 33) and (**d**) *ABCC4* (median expression-28.4; below median n = 30 vs above median n = 26) RNA. The RNA expression of each gene was normalised to GAPDH and relative expression to CML001 using 2^-ddCT^ method. Statistical significance was calculated using Mann–Whitney U test.

When the role of genetic variants in the target imatinib metabolism/ transport genes on trough plasma imatinib levels was evaluated, patients with variant MDR1/*ABCB1*-C1236T had significantly high day29 plasma imatinib concentration (p = 0.005) compared to those with wild type genotype (Fig. 2a). None of the other variants were significantly associated with plasma imatinib levels.

### RNA expression of ABC transporter genes significantly influence intracellular but not plasma imatinib levels

The expression of influx and efflux transporters was compared with plasma and intracellular imatinib levels. Patients who had *ABCB1* expression above median showed significantly lower intracellular imatinib levels (p = 0.0293) (Fig. 2b). Patients who had *ABCA3* and *ABCC4* expression above median had lower intra cellular imatinib levels (*ABCA3* p = 0.0747; *ABCC4* p = 0.0536) (Fig. 2c, d).

### RNA expression of imatinib transporter genes in the CD34^+^ fraction and response to therapy

**Figure 3:**
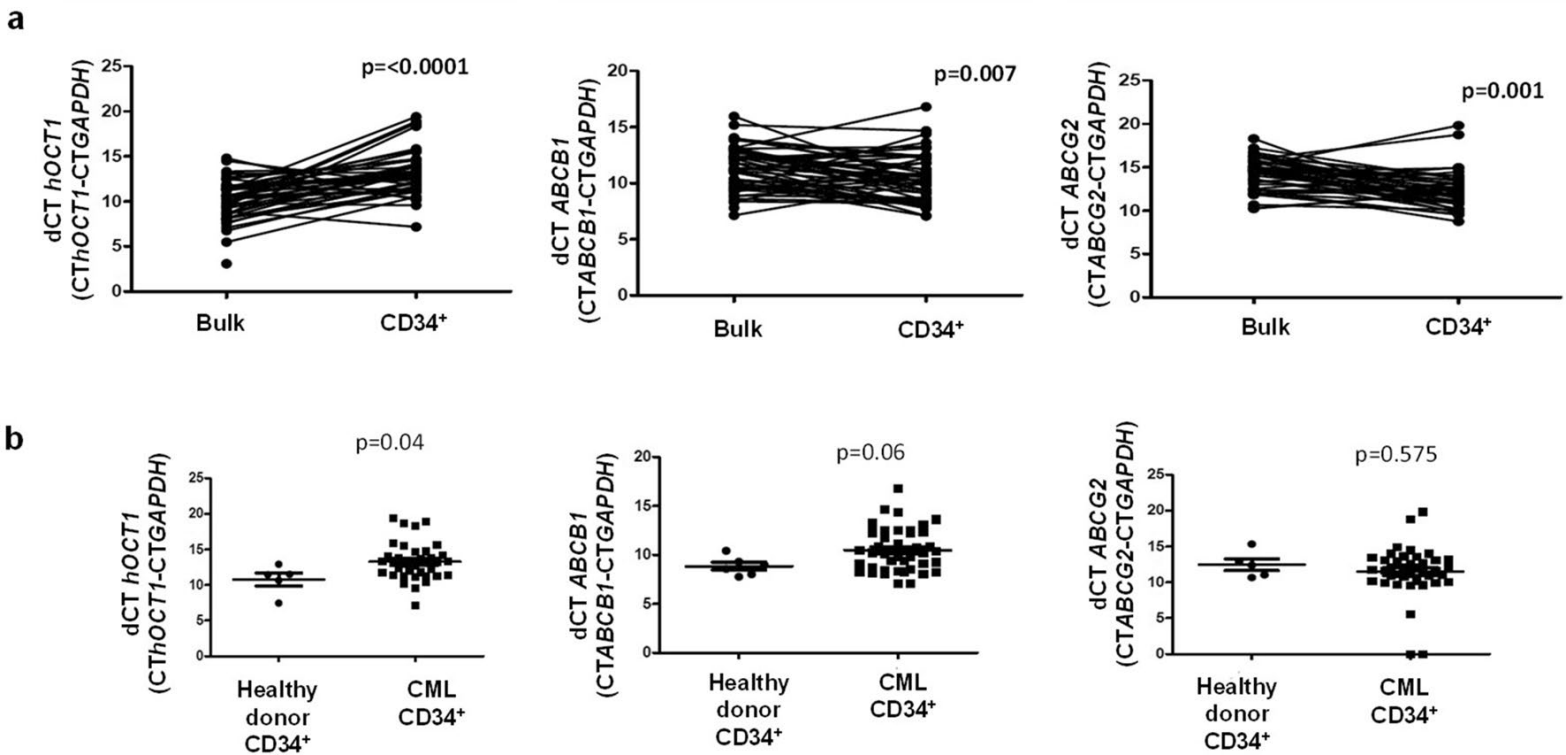
Expression of imatinib influx and efflux transporters in bulk Vs CD34^+^ primary CML cells. (**a**) Expression of *hOCT1*, *ABCB1*/*MDR1* and *ABCG2* in CML bulk cells compared with expression of transporter expression in CML CD34 + (Leukemic stem cells) LSCs cells. Statistical significance was calculated using paired-T test. (**b**) Expression of *hOCT1*, *ABCB1*/*MDR1* and *ABCG2* in CD34^+^ cells from normal healthy donors vs. primary CML CD34^+^ cells where higher the dCt lower the expression and vice versa. Statistical significance was calculated using Mann–Whitney U test.

The expression of Imatinib efflux (*ABCB1*, *ABCG2)* and influx (*SLC22A1)* transporters in CD34^+^ as well as bulk CML cells was compared. There was a significantly increased expression of efflux transporters *ABCG2* (p = 0.001) and *ABCB1* (p = 0.007) and decreased expression of *SLC22A1* (p =  < 0.001) in CD34^+^ fraction compared to the total cellular RNA (Fig. 3a). Decreased expression of *hOCT-1* mRNA was observed in CML CD34^+^ cells compared to CD34^+^ cells derived from normal healthy donors (Fig. 3b). There was no significant association between RNA expression of these transporters in the CD34^+^ cells with EMR and MMR.

### Genetic polymorphisms in imatinib transporter and drug metabolizing genes influence EMR and MMR to imatinib therapy

We further analyzed the influence of genetic polymorphisms in target genes on the incidence of EMR at 3 & 6 months and MMR at 12 months. The incidence of EMR at 3 months was significantly higher in patients with *MDR1*-C1236T variant genotype ((p = 0.044) Table 3) compared to those with wild type genotype. The incidence of EMR at 6 months was higher in patients with *MDR1*-C3435T variant genotype (p = 0.058) (Table 3) compared to those with wild type genotype. Also, the incidence of EMR at 6 months was higher in patients with *GSTM1* wild type genotype compared to those with null genotype (p = 0.071).

**Table 3:** *ABCB1*/*MDR1* C1236T, C3435T and *GSTM1* genotype influences early molecular response at 3 and 6 months. Statistical significance was calculated using Fisher exact test.

	Genotype	*BCR-ABL1* < 10% n (%)	*BCR-ABL1* > 10% n (%)	*p* value
*MDR1* 1236 genotype & EMR at 3 months (n = 114)	Wt	7 (35)	13 (65)	0.045*
	Het/Mut	58 (61.7)	36 (38.2)	

	Genotype	*BCR-ABL1* < 1% n (%)	*BCR-ABL1* > 1% n (%)	*p* value
*MDR1* 3435 genotype & EMR at 6 months (n = 134)	Wt	6 (28.5)	15 (71.5)	0.058
	Het/Mut	59 (52.2)	54 (47.8)	
*GSTM1* genotype & EMR at 6 months (n = 134)	Positive	47 (55.3)	38 (44.7)	0.071
	Negative	18 (38.3)	29 (61.7)	

### Factors influencing 2-year FFS post imatinib therapy

**Figure 4:**
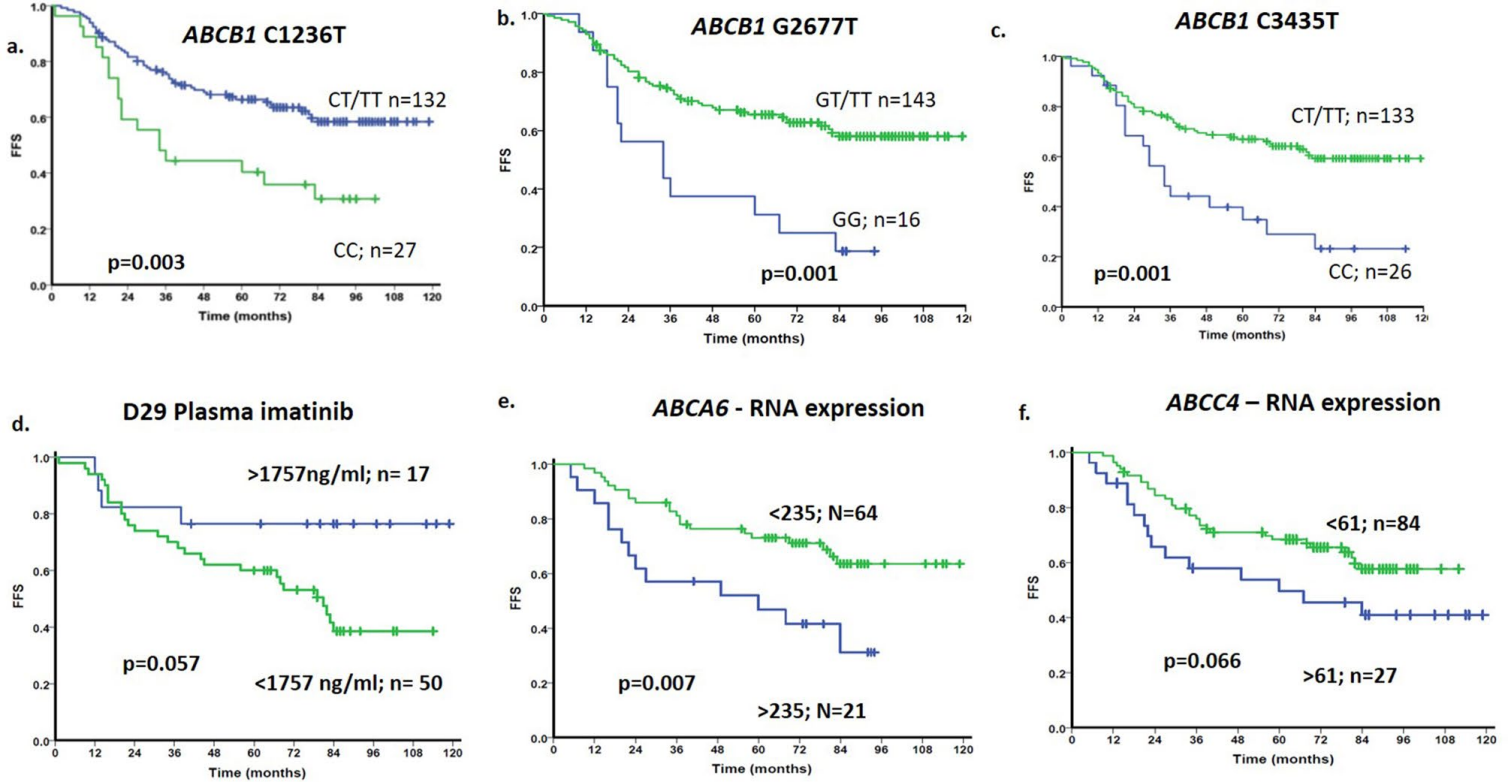
*MDR1* polymorphisms, plasma imatinib levels, and RNA expression of efflux transporters influence FFS after imatinib therapy in patients with CP-CML. (a)–(**c**) Influence of *MDR1* genotypes C1236T, G2677T & C3435T on FFS after imatinib therapy. (**d**) d. Influence of plasma imatinib levels on day29 on FFS. (**e**)–(**f**) Influence of *ABCA6* and *ABCC4* expression on FFS after imatinib therapy. RNA expression was normalised to *GAPDH* as the housekeeping gene and expressed relative to the expression in CML001 using 2^-ddCT^ method.

We further analyzed the influence of all the basic demographic (including age, sex, Sokal score, *BCR-ABL1* transcript type) as well as variables including imatinib plasma levels, RNA expression and genetic polymorphisms of imatinib influx/efflux transporters on 2-year FFS post imatinib therapy. Patients with low (< 0.8) and intermediate (0.8–1.2) Sokal score showed better 2-year FFS compared to those with high Sokal score (> 1.2) (40/110 vs. 28/50 patients with low/intermediate vs. high Sokal score respectively had failure to imatinib therapy; p = 0.02). None of the other basic demographic factors was significantly associated with FFS or EMR. Patients who had *MDR1* variant genotype showed significantly better FFS compared to the wild type genotype (*MDR1*- 1236; p = 0.005, *MDR1*- 2677; p = 0.004 and *MDR1*- 3435; p = 0.004) (Fig. 4a–c). Also, patients with higher median Imatinib levels (> 1757 ng/mL) on day29 had significantly better FFS (p = 0.057) (Fig. 4d). Patients with *ABCA6* expression below median showed significantly better FFS compared to those with above median levels (p = 0.007) (Fig. 4e). There was a trend to significantly lower rate of FFS in patients with *ABCC4* expression above median compared to below median (p = 0.066) (Fig. 4f.). Patients who achieved EMR at 3 and 6 months had significantly better rate of FFS compared to those who did not achieve EMR (Supplementary Fig. 1a-b). Patients who achieved MMR at 12 months also showed significantly better rate of FFS compared to those who did not achieve MMR (p = 0.002) (Supplementary Fig. 1c). High Sokal score (Hazard ratio: 1.53; *p* value: 0.05), *MDR1* C2677T genotype (Hazard ratio: 0.401; *p* value: 0.014) and not achieving EMR at 3 months post imatinib therapy (Hazard ratio: 3.875; *p* value: 0.0001) were significant risk factors for 2-year FFS in multivariate analysis (Tables 4, 5). Plasma imatinib levels were available only in 51 patients for whom molecular response was also available and hence could not be included as a variable in multivariate analysis.

**Table 4:** Univariate and Multivariate analysis for 2-year failure-free survival.

Variables	*p* value	Hazard ratio
Sokal score	0.001*	1.835 (1.272–2.647)
EMR at 3 months	0.000*	4.092 (2.269–7.381)
EMR at 6 months	0.000*	6.291 (3.281—12.061)
MMR at 12 months	0.000*	10.627 (4.552–24.809)
Desmethyl imatinib day-29	0.052	0.997(0.993–1.000)
plasma imatinib day-29	0.010*	0.999 (0.999–1.000)
ABCA6	0.009*	0.399 (0.199–0.799)
ABCC1	0.055	1 (1.000–1.011)
ABCC4	0.070	0.567 (0.307–1.048)
MDR1 G2677T	0.001*	0.374 (0.204–0.686)
MDR1 C3435T	0.002*	0.416 (0.242–0.715)
Sokal score	0.05*	1.513 (1.001- 2.287)
ABCB1 (MDR1) C2677T variant genotype	0.014*	0.401 (0.193–0.833)
Not achieving EMR at 3 months	0.0001*	3.875 (2.132–7.046)

**Table 5:** Association of steady state plasma imatinib levels with response- comparison with previous studies.

Reference	N	Response	Responder		Non responders		*p* value
			N	Mean C0 ng/mL	N	Mean C0 ng/mL	
Larson et al., 2008^16^	351	CCyR	297	1099 ± 554	54	812 ± 409	0
Takahashi et al., 2010^51^	254	CCyR	218	1057 ± 585	36	835 ± 524	0.033
		MMR	166	1107 ± 594	88	873 ± 528	0.002
Picard et al., 2007^17^	68	CCyR	56	1123 ± 617	12	694 ± 556	0.03
		MMR	34	1452 ± 649	34	869 ± 427	0.001
Ishikawa et al., 2010^26^	60	MMR	38	1093	22	853	0.002
				562–2150		361–1205	
Forrest et al., 2009^52^	78	CCyR	53	1010 ± 469	24	1175 ± 656	0.29
		MMR	51	1067 ± 473	27	1063 ± 643	0.74
Singh et al., 2009^53^	40	Clinical response	20	2340 ± 520	20	690 ± 150	0.002
Malhotra et al., 2014^43^	131	Molecular response (< 1%)	104	2110 ± 1180	27	1310 ± 720	0.001
Natarajan et al. 2019^54^	173	MMR	71	2333 ± 1112	102	1643 ± 1384	P < 0.001
Arora et al., 2013^44^	46	CCyR	NA	2157 ± 1287	NA	1884 ± 809	P > 0.05
Present study	51	EMR@3 months	28	1532 ± 939	23	971 ± 714	0.022*
	65	MMR@12 months	28	1566 ± 991	37	1160 ± 921	0.042*

## Discussion

Targeted therapy with imatinib is still the first line treatment of choice in newly diagnosed patients with CML in chronic phase (CML-CP). However, a proportion of patients do not achieve milestone molecular responses with imatinib and are classified as sub-optimal responders. Identifying these patients followed by early switch of therapy will help improve FFS. Although several studies have evaluated factors influencing attainment of MMR after imatinib therapy, to the best of our knowledge, there is no prospective study evaluating various factors influencing EMR and FFS after imatinib therapy, especially in a uniform cohort of CP-CML patients. We conducted a prospective single centre observational study in CP-CML patients with serial molecular monitoring and comprehensive pharmacogenetic analysis to evaluate factors influencing EMR to imatinib and FFS. Response to imatinib as seen by achieving milestone molecular response post imatinib therapy was similar to previous reports^35,36^^.^ Mutations in the *BCR-ABL1* kinase domain explained only 28% of the patients with sub-optimal response, which is also similar to previous reports^37–39^. None of the basic demographic parameters including age, sex, Sokal score and *BCR-ABL1* transcript type showed significant association with achieving EMR/MMR post imatinib therapy in the present study. Similar to previous reports from India, the median age of diagnosis in of CML in our cohort is lower compared to Western patients (36 yrs vs. 47yrs)^40^, majority of the patients younger than 40 years. This is probably the reason for age not showing significant association with response to imatinib therapy. While the distribution of low, intermediate and high Sokal scores was similar to Western data in CP-CML patients as reviewed by Ganesan and Kumar^40^, patients with high Sokal score showed significantly poor 2-year FFS in this study, similar to previous reports^41,42^.

Several studies have reported that genetic variants in the influx and efflux transporters of imatinib namely *hOCT1*, *ABCG2* and *MDR1* to be associated with imatinib levels^16,20,43,44^. In the present study, similar to previous reports, patients with variant *MDR1* genotype showed significant association with plasma imatinib levels. The variants of *MDR1* C1236T, C3435T and G2326T were shown to result in decreased *MDR1* expression^45–47^ which in turn results in higher plasma imatinib levels and hence better EMR/MMR/FFS. Increased RNA expression of ABC transporters *ABCB1*, *ABCA3* and *ABCC4* were significantly associated with lower intracellular imatinib levels in our study. It has been reported previously that increased expression of *ABCB1*^48^; *ABCA3*^49^ and *ABCC4*^50^ to be associated with imatinib resistance. Unlike previous reports, none of the genetic variants in *hOCT1* were significantly associated with plasma imatinib levels or molecular response in the present study.

Higher trough plasma Imatinib levels have been shown to be associated with significantly better molecular response (Table 4), including the present study. The median plasma imatinib levels in patients with good response vs. suboptimal response in our study is similar to previous reports^16,17,26,51,52^. However, from the available data from India, although various measures of response including clinical, cytogenetic or MMR were considered, the imatinib levels in good responders seems to be higher than the previous reports from outside India^43,44,53,54^, and the present study. The two Indian studies that showed association between molecular response and plasma Imatinib levels have assessed either hematological response^43,53^ or considered *BCR-ABL1* ratio below 1% as molecular response. The median trough plasma Imatinib levels on day29 can be a used as biomarker for assessment of response to imatinib therapy to rule out issues related to poor compliance as well as identifying poor or ultra-rapid metabolizers who could benefit from adjustment of imatinib doses.

RNA expression of influx and efflux transporters of imatinib have been shown to influence response to imatinib therapy. Several studies have reported increased expression or functional activity of *hOCT1* to be associated with better response to imatinib^13,14,55^. However, the RNA expression of these transporters did not show any association with EMR or MMR in the present study. Interestingly, when the expression of these transporters in CML CD34^+^ vs. bulk cells was compared, there was significantly increased *ABCB1* and *ABCG2* expression and decreased *hOCT1* in the CD34 + cells compared to CML bulk cells. This is in line with the fact that imatinib does not eliminate CML stem cells^56,57^ probably due to this dysregulated expression of the transporters in the CML CD34^+^ cells.

Polymorphisms in imatinib influx/ efflux transporters have been reported to influence response to imatinib therapy and progression free survival^15,19,58–60^. Similar to these reports, *MDR1* and *ABCG2* variants were associated with better EMR/MMR in the present study. Higher plasma imatinib levels^16,60^, attainment of EMR (at 3 or 6 months)^61–63^ & MMR at 12 months^64,65^ have been reported to result in better FFS in patients with CML on imatinib therapy. In the present study, *MDR1* variants, day29 plasma imatinib level of > 1757 ng/mL, lower *ABCA6*, *ABCC4* RNA expression as well as achieving EMR at 3, 6 months and MMR at 12 months were associated with significantly better FFS. While increased *ABCC4* RNA expression has been reported previously in suboptimal response to imatinib^15,17,21,22,26^, the role on increased *ABCA6* on imatinib response is not clear.

Our study suggests that factors such as steady state plasma Imatinib levels, *MDR1* polymorphisms and ABC transporter expression influence EMR/MMR to imatinib therapy, which in turn influence FFS in patients with CP-CML. The possibility to tailor dose of imatinib considering these factors in order to improve molecular response to imatinib and better FFS, remains to be tested.

## Supplementary information

Supplementary Figure 1.

Supplementary Tables.
