# Supplementary material for: Plasma imatinib levels and ABCB1 polymorphism influences early molecular response and failure-free survival in newly diagnosed chronic phase CML patients
Source: Sci Rep. 2020 Nov 26;10:20640. doi: 10.1038/s41598-020-77140-9 (PMC7691501; doi:10.1038/s41598-020-77140-9)
Supplement: Supplementary file 1 — Supplementary Figure 1. [file 41598_2020_77140_MOESM1_ESM.pptx]

## Slide 1
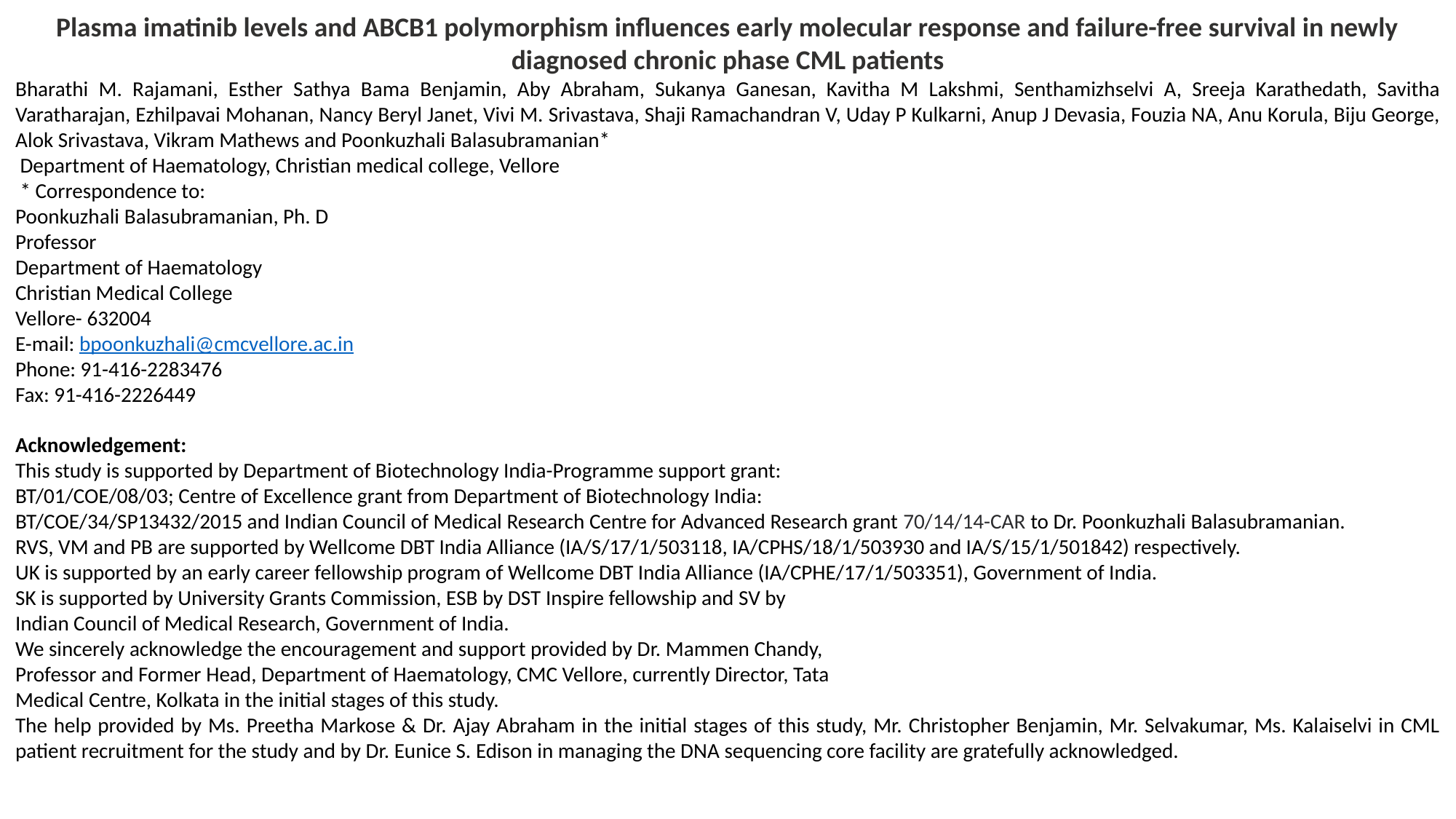

Plasma imatinib levels and ABCB1 polymorphism influences early molecular response and failure-free survival in newly diagnosed chronic phase CML patients
Bharathi M. Rajamani, Esther Sathya Bama Benjamin, Aby Abraham, Sukanya Ganesan, Kavitha M Lakshmi, Senthamizhselvi A, Sreeja Karathedath, Savitha Varatharajan, Ezhilpavai Mohanan, Nancy Beryl Janet, Vivi M. Srivastava, Shaji Ramachandran V, Uday P Kulkarni, Anup J Devasia, Fouzia NA, Anu Korula, Biju George, Alok Srivastava, Vikram Mathews and Poonkuzhali Balasubramanian*
 Department of Haematology, Christian medical college, Vellore
 * Correspondence to:
Poonkuzhali Balasubramanian, Ph. D
Professor
Department of Haematology
Christian Medical College
Vellore- 632004
E-mail: bpoonkuzhali@cmcvellore.ac.in
Phone: 91-416-2283476
Fax: 91-416-2226449
Acknowledgement:
This study is supported by Department of Biotechnology India-Programme support grant:
BT/01/COE/08/03; Centre of Excellence grant from Department of Biotechnology India:
BT/COE/34/SP13432/2015 and Indian Council of Medical Research Centre for Advanced Research grant 70/14/14-CAR to Dr. Poonkuzhali Balasubramanian.
RVS, VM and PB are supported by Wellcome DBT India Alliance (IA/S/17/1/503118, IA/CPHS/18/1/503930 and IA/S/15/1/501842) respectively.
UK is supported by an early career fellowship program of Wellcome DBT India Alliance (IA/CPHE/17/1/503351), Government of India.
SK is supported by University Grants Commission, ESB by DST Inspire fellowship and SV by
Indian Council of Medical Research, Government of India.
We sincerely acknowledge the encouragement and support provided by Dr. Mammen Chandy,
Professor and Former Head, Department of Haematology, CMC Vellore, currently Director, Tata
Medical Centre, Kolkata in the initial stages of this study.
The help provided by Ms. Preetha Markose & Dr. Ajay Abraham in the initial stages of this study, Mr. Christopher Benjamin, Mr. Selvakumar, Ms. Kalaiselvi in CML patient recruitment for the study and by Dr. Eunice S. Edison in managing the DNA sequencing core facility are gratefully acknowledged.

## Slide 2
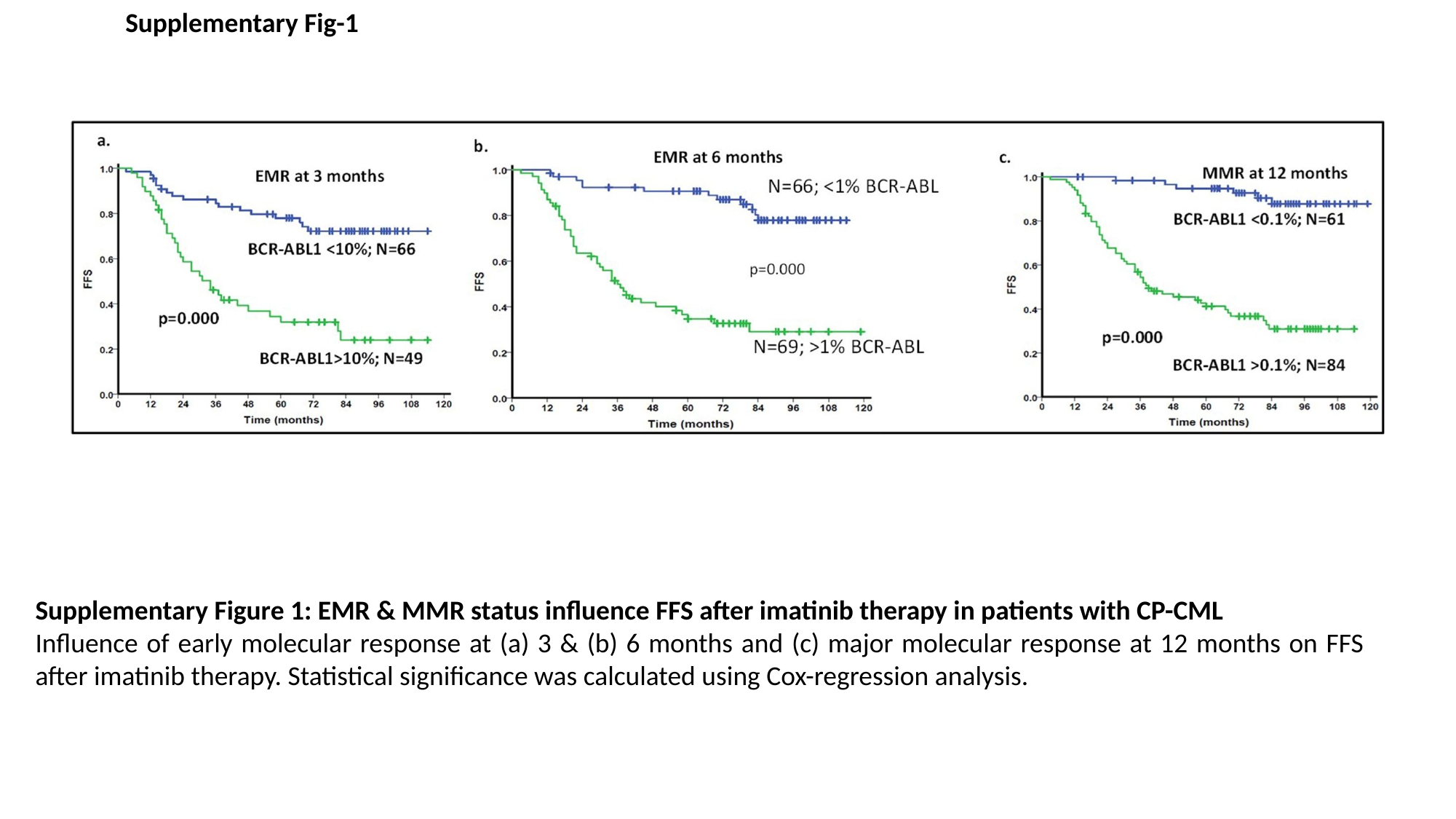

Supplementary Fig-1
Supplementary Figure 1: EMR & MMR status influence FFS after imatinib therapy in patients with CP-CML
Influence of early molecular response at (a) 3 & (b) 6 months and (c) major molecular response at 12 months on FFS after imatinib therapy. Statistical significance was calculated using Cox-regression analysis.
