# Supplementary material for: Plasma imatinib levels and ABCB1 polymorphism influences early molecular response and failure-free survival in newly diagnosed chronic phase CML patients
Source: Sci Rep. 2020 Nov 26;10:20640. doi: 10.1038/s41598-020-77140-9 (PMC7691501; doi:10.1038/s41598-020-77140-9)
Supplement: Supplementary file 2 — Supplementary Tables. [file 41598_2020_77140_MOESM2_ESM.docx]

**Plasma imatinib levels and *ABCB1* polymorphism influences early molecular response and failure-free survival in newly diagnosed chronic phase CML patients**

Bharathi M. Rajamani, Esther Sathya Bama Benjamin, Aby Abraham, Sukanya Ganesan, Kavitha M Lakshmi, Senthamizhselvi A, Sreeja Karathedath, Savitha Varatharajan, Ezhilpavai Mohanan, Nancy Beryl Janet, Vivi M. Srivastava, Shaji Ramachandran V, Uday P Kulkarni, Anup J Devasia, Fouzia NA, Anu Korula, Biju George, Alok Srivastava, Vikram Mathews and Poonkuzhali Balasubramanian*

Department of Haematology, Christian medical college, Vellore

* Correspondence to:

Poonkuzhali Balasubramanian, Ph. D

Professor

Department of Haematology

Christian Medical College

Vellore- 632004

E-mail: [bpoonkuzhali@cmcvellore.ac.in](mailto:bpoonkuzhali@cmcvellore.ac.in)

Phone: 91-416-2283476

Fax: 91-416-2226449

**Acknowledgement:**

This study is supported by Department of Biotechnology India-Programme support grant:

BT/01/COE/08/03; Centre of Excellence grant from Department of Biotechnology India:

BT/COE/34/SP13432/2015 and Indian Council of Medical Research Centre for Advanced Research grant 70/14/14-CAR to Dr. Poonkuzhali Balasubramanian.

RVS, VM and PB are supported by Wellcome DBT India Alliance (IA/S/17/1/503118, IA/CPHS/18/1/503930 and IA/S/15/1/501842) respectively.

UK is supported by an early career fellowship program of Wellcome DBT India Alliance (IA/CPHE/17/1/503351), Government of India.

SK is supported by University Grants Commission, ESB by DST Inspire fellowship and SV by

Indian Council of Medical Research, Government of India.

We sincerely acknowledge the encouragement and support provided by Dr. Mammen Chandy,

Professor and Former Head, Department of Haematology, CMC Vellore, currently Director, Tata

Medical Centre, Kolkata in the initial stages of this study.

The help provided by Ms. Preetha Markose & Dr. Ajay Abraham in the initial stages of this study, Mr. Christopher Benjamin, Mr. Selvakumar, Ms. Kalaiselvi in CML patient recruitment for the study and by Dr. Eunice S. Edison in managing the DNA sequencing core facility are gratefully acknowledged.

**Supplementary Table 1: List of primers used for screening the SLC22A1, ABCB1, ABCG2,**

**CYP3A4/A5, OCTN1, GSTM1/T1/P1 and BIM genetic variants**

| **Gene** | **Exon** | **Primers (5'-3')** | **Length (bp)** | **Anne Temp** |
| --- | --- | --- | --- | --- |
| *hOCT1/SLC22A1* | Exon1F | TGAGGGAGACATT | 559 | 57°C |
|  | Exon1R | GGAACTGAACTTCATAGGATT |  |  |
|  | Exon2F | AGTCCTGACTCACACATGGTTC | 240 | 59°C |
|  | Exon2R | TGAGAACAGATTCGCCCTTAG |  |  |
|  | Exon 5-6F | GGCTGAACGTCAC | 553 | 57°C |
|  | Exon 5-6R | AATCTATAGCCCA |  |  |
|  | Exon7F | TTTCTTCAGTCTCT | 322 | 59°C |
|  | Exon7R | TCCCCACACTTCG |  |  |
| *MDR1*/*ABCB1* | Exon 12F | TATCCTGTGTCTGTGAATTGCC | 366 | 51.5°C |
|  | Exon12R | CCTGACTCACCACACCAATG |  |  |
|  | Exon21F | TGCAGGCTATAGGTTCCAGG | 244 | 55°C |
|  | Exon21R | TTTAGTTTGACTCACCTTCCCG |  |  |
|  | Exon26F | TGT TTT CAG CTG CTT GAT GG | 197 | 55°C |
|  | Exon26R | AAG GCA TGT ATG TTG GCC TC |  |  |
| *ABCG2* | Pro-F | GACGCAATCATGTAGAACATAACA | 1184 | 61°C |
|  | Pro-R | ATGGACAGCAGTGTGTCCTTGAGA |  |  |
|  | Exon2F | ATGGTATGGGCCATTCATTG | 497 | 56°C |
|  | Exon2R | TGGCCCAGTTATTTCACTCC |  |  |
|  | Exon5F | GCAGAACTGCAGGTTCATCAT | 372 | 60°C |
|  | Exon5R | TGGAAAGCAACCATTTTTGA |  |  |
| *Cyp3A4**1B | Pro-F | GGACAGCCATAGAGACAAGGGG | 168 | 59°C |
|  | Pro-R | CACTCACTGACCTCCTTTGAGTTCA |  |  |
| *Cyp3A5**3 | Intron3F | CATCGTTAGTAGACAGATGA | 293 | 55°C |
|  | Intron3R | GGTCCAAACAGGGAAGAAATA |  |  |
| *Cyp3A5**6 | Exon7F | GTGGGGTGTTGACAGCTAAAG | 495 | 55°C |
|  | Exon7R | TGGAAGATGATTCAGCAGATAGT |  |  |
| *GSTM1* | F | GAACTCCCTGAAAAGCTAAAGC | 219bp | 62°C |
|  | R | GTTGGGCTCAAATATACGGTGG |  |  |
| *GSTT1* | F | TTCCTTACTGGTCCTCACATCTC | 459bp | 62°C |
|  | R | TCACCGGATCATGGCCAGCA |  |  |
| Albumin | F | GCCCTCTGCTAACAAGTCCTAC | 350bp | 62°C |
|  | R | GCCCTAAAAAGAAAATCGCCAAT |  |  |
| *BIM* | Wt F | ACTGTAAAACGACGGCCAGTCCTCATGATGAAGGCTAACTCAA | 213bp | 60°C |
|  | Wt R | ACCAGGAAACAGCTATGACCAACCTCTGACAAGTGACCACCA |  |  |
|  | Del F | ACTGTAAAACGACGGCCAGTCCTCATGATGAAGGCTAACTCAA | 173bp | 60°C |
|  | Del R | ACCAGGAAACAGCTATGACCGGCACAGCCTCTATGGAGAACA |  |  |
| *OCTN1* | F | TGTTCTTATGTCCCGGGCTT | 302bp | 52°C |
|  | R | GCCCAGCCAACAATATGCTT |  |  |
| *GSTP1* | F | CTTTCTTTGTTCAGCCCCCA | 218bp | 58°C |
|  | R | ATCCCCAGTGACTGTGTGTGTT |  |  |

**Supplementary Table-2: BCR-ABL kinase domain mutation in imatinib sub-optimal responders:**

| Mutation screened | N=79 |  |
| --- | --- | --- |
| Mutation detected | N=22 |  |
|  | T315I= 5  G250E=5  E255K=2  F359I=2 | E281K, E292G, F359V, L248V, L387M, del aa362-386, H246H, T267T = 1 each |

In patients with sub-optimal response to imatinib at 3, 6 or 12 months, mutation in *BCR-ABL1* kinase domain was screened by Sanger Sequencing. Of the 57 patients with no mutation, 13 switched to nilotinib therapy.

**Supplementary table-3: Genotype frequency of transporter and metabolizing enzyme gene variants in patients with CML (n=159)**

| db SNP ID | Gene | Location | Nucleotide change | AA change | Genotype Frequency | | |
| --- | --- | --- | --- | --- | --- | --- | --- |
|  |  |  |  |  | wt | het | mut |
| rs1867351 | *hOCT1* | Exon 1 | C>T | Ser51Ser | 0.63 | 0.32 | 0.05 |
| rs12208357 | *hOCT1* | Exon 1 | C>T | Arg61Cys | 0.925 | 0.068 | 0.07 |
| rs683369 | *hOCT1* | Exon 2 | C>G | Leu160Phe | 0.724 | 0.239 | 0.037 |
| rs201942835 | *hOCT1* | Exon2 | G>T | Gly165Cys | 0.91 | 0.09 | - |
| rs4646277 | *hOCT1* | Exon5 | C>T | Pro283Leu | 1 | - | - |
| rs4646278 | *hOCT1* | Exon5 | C>T | Arg287Gly | 0.985 | 0.015 | - |
| rs77092743 | *hOCT1* | Intron 5 | G>A | Non-coding | 0.694 | 0.284 | 0.022 |
| rs7762846 | *hOCT1* | Intron5 | C>T | Non-coding | 0.813 | 0.165 | 0.022 |
| Novel | *hOCT1* | Exon6 | C>T | Thr340Met | 0.992 | 0.008 | - |
| rs2282143 | *hOCT1* | Exon6 | C>T | Pro341Leu | 0.695 | 0.283 | 0.022 |
| rs628031 | *hOCT1* | Exon7 | A>G | Met408Val | 0.105 | 0.41 | 0.485 |
| rs72552763 | *hOCT1* | Exon7 | >GAT | Met420-Ile421 | 0.007 | 0.296 | 0.724 |
| rs4646281 | *hOCT1* | Intron7 | >ins | Non-coding | 0.105 | 0.41 | 0.485 |
| rs9457843 | *hOCT1* | Intron7 | C>T | Non-coding | 0.825 | 0.14 | 0.035 |
| rs1128503 | *ABCB1* | Exon 12 | C>T | Gly412Gly | 0.208 | 0.459 | 0.333 |
| rs2032582 | *ABCB1* | Exon 21 | G>T | Ala893Ser | 0.148 | 0.496 | 0.356 |
| rs1045642 | *ABCB1* | Exon 26 | C>T | Ile1145Ile | 0.2 | 0.496 | 0.304 |
| rs2231137 | *ABCG2* | Exon 2 | G>A | Val12Met | 0.667 | 0.274 | 0.059 |
| rs2231142 | *ABCG2* | Exon 5 | C>A | Gln141Lys | 0.844 | 0.134 | 0.02 |
| rs7699188 | *ABCG2* | Promoter | C>T | Non-coding | 0.585 | 0.356 | 0.059 |
| rs2740574 | *CYP3A4**1B | Promoter | A>G | Non-coding | 0.889 | 0.104 | 0.007 |
| rs776746 | *CYP3A5**3 | Intron 3 | A>G | Non-coding | 0.133 | 0.474 | 0.393 |
| rs10264272 | *CYP3A5**6 | Exon 7 | C>T | Lys208Lys | 1 | - | - |
| rs1050152 | *OCTN1* | Exon 9 | C>T | Leu411Phe | 0.902 | 0.0978 | 0 |
| rs3957357 | *GSTA1* | Promoter | T>G | Non-coding | 0.5543 | 0.3586 | 0.0869 |
| rs1695 | *GSTP1* | Exon 5 | A>G | Ile105Val | 0.4969 | 0.411 | 0.092 |
|  | *GSTT1* |  | >del |  | 0.685 | NA | 0.315 |
|  | *GSTM1* |  | >del |  | 0.819 | NA | 0.181 |
|  | *BIM* |  | >del |  | 1 | NA | 0 |

**Supplementary table-4: Genotypic frequency of *hOCT1/SLC22A1* in normal healthy volunteers (n=100)**

| db SNP ID | Gene | Location | Nucleotide change | AA change | Genotype Frequency in normal controls | | |
| --- | --- | --- | --- | --- | --- | --- | --- |
|  |  |  |  |  | wt | het | mut |
| rs1867351 | *SLC22A1* | Exon 1 | C>T | Ser51Ser | 0.646 | 0.313 | 0.042 |
| rs12208357 | *SLC22A1* | Exon 1 | C>T | Arg61Cys | 0.958 | 0.042 | 0 |
| rs683369 | *SLC22A1* | Exon 2 | C>G | Leu160Phe | 0.573 | 0.396 | 0.031 |
| rs201942835 | *SLC22A1* | Exon2 | G>T | Gly165Cys | 0.958 | 0.042 | 0 |
| rs4646277 | *SLC22A1* | Exon5 | C>T | Pro283Leu | 1 | 0 | 0 |
| rs4646278 | *SLC22A1* | Exon5 | C>T | Arg287Gly | 0.99 | 0.01 | 0 |
| rs77092743 | *SLC22A1* | Intron 5 | G>A | noncoding | 0.896 | 0.104 | 0 |
| rs7762846 | *SLC22A1* | Exon5 | C>T | noncoding | 0.833 | 0.167 | 0 |
| Novel | *SLC22A1* | Exon6 | C>T | Thr340Met | 0.969 | 0.031 | 0 |
| rs2282143 | *SLC22A1* | Exon6 | C>T | Pro341Leu | 0.875 | 0.125 | 0 |
| rs628031 | *SLC22A1* | Exon7 | A>G | Met408Val | 0.177 | 0.479 | 0.344 |
| rs72552763 | *SLC22A1* | Exon7 | del>GAT | Met420-Ile421 | 0.75 | 0.208 | 0.042 |
| rs4646281 | *SLC22A1* | Intron7 | del>ins | noncoding | 0.177 | 0.5 | 0.323 |
| rs9457843 | *SLC22A1* | Intron7 | C>T | noncoding | 0.844 | 0.073 | 0 |

**Supplementary table-5: RNA expression of imatinib influx transporter *hOCT1* and ABC transporters in primary CML cells from patients before the start of TKI therapy. RNA expression was normalised to housekeeping gene *GAPDH* and the results were normalized with reference to CML patient-001.**

| **Gene name** | **median** | **Range** |
| --- | --- | --- |
| *hOCT1* | 21.06 | 0.62-4389 |
| *MDR1/ABCB1* | 21.9 | 1.79-492 |
| *ABCG2* | 208.76 | 19.72-5260 |
| *ABCA3* | 505.02 | 7.11-44470 |
| *ABCA5* | 48.32 | 16.3-390 |
| *ABCA6* | 113 | 12-4663 |
| *ABCB5* | 73.34 | 5.68-1726 |
| *ABCB6* | 74.69 | 15.87-1821 |
| *ABCB7* | 43.78 | 17.34-340 |
| *ABCB8* | 55.87 | 17.05-634 |
| *ABCB10* | 64.38 | 31.4-260 |
| *ABCB11* | 30.9 | 8.6-339 |
| *ABCC1* | 45.5 | 16.5-282 |
| *ABCC3* | 31.5 | 1.78-1613 |
| *ABCC4* | 28.49 | 5.82-1156 |
| *ABCC11* | 13.2 | 2.52-122 |

**Supplementary table-6: Number of samples for which data was available for each variable**

| **Variable** | **Data available n/160** |
| --- | --- |
| EMR status@ 3 months | 116* |
| EMR status@ 6 months | 135* |
| MMR @ 12 months | 145* |
| Genetic polymorphisms | 159 ^#^ |
| BIM deletion | 159 ^#^ |
| Plasma imatinib levels (Day29) | 67* |
| Plasma imatinib levels and 3 months EMR | 51 |
| Plasma imatinib levels and 6 months MMR | 57 |
| Plasma imatinib levels and 12 months MMR | 65 |
| Intracellular IM | 64* |
| *MDR1, hOCT1, ABCG2 & ABCA3* RNA expression | 134^¥^ |
| *ABCA5*, *ABCA6*, *ABCB5*, *ABCB7*, *ABCB8*, *ABCB10*, *ABCB11* RNA expression | 85^¥^ |
| *ABCB6* RNA expression | 109^¥^ |
| *ABCC1*, *ABCC3*, *ABCC4* RNA EXPRESSION | 111^¥^ |
| *MDR1*, *hOCT1* & *ABCG2* RNA expression in CML CD34+ cells | 39^£^ |

Note:

^¥^data not available for remaining patients due to non-availability of RNA.

^#^DNA sample not available for one patient due to severe cytopenia.

*Molecular response, plasma imatinib levels and intra cellular imatinib levels were not available for the remaining patients due to loss of follow up.

£CD34 enrichment was done only for few patients.
